# Development and Validation of a Wearable Softness Sensor Based on Fingernail Deformation and an Inertial Measurement Unit for Quantifying Palpation

**DOI:** 10.7759/cureus.104461

**Published:** 2026-02-28

**Authors:** Shohei Ueda, Hiroya Fukuda

**Affiliations:** 1 Graduate School of Human Development and Environment, Kobe University, Kobe, JPN

**Keywords:** fingernail sensor, inertial measurement unit, palpation, stiffness sensing, tactile sensor, wearable sensor

## Abstract

Introduction

Palpation plays a crucial role in diagnosis; however, the technique relies heavily on subjectivity, presenting challenges for quantitative instruction in medical education. Many existing palpation sensors cover the fingertip, thereby obstructing the tactile sensation essential for palpation. Therefore, in this study, we aimed to develop and evaluate a wearable softness sensor that does not obstruct tactile sensation, with a view toward its application in medical education.

Methods

The proposed sensor system consists of fingertip force estimation using fingernail strain and indentation depth estimation using an inertial measurement unit (IMU). Accuracy verification experiments were conducted for each estimation method, followed by a softness discrimination experiment using three types of sponges with varying levels of softness.

Results

The results demonstrated that the proposed sensor achieved high estimation accuracy for both fingertip force (root mean square error (RMSE): 0.17 N) and displacement (RMSE: 0.75 mm). Furthermore, the system successfully and significantly discriminated the stiffness values of the three sponge types, with the estimated values showing good agreement with reference values measured using a force gauge.

Conclusion

The proposed sensor enables the quantification of object softness without interfering with natural palpation movements. These findings demonstrate the system's potential as a tool for the objective evaluation of palpation skills and as an effective training device.

## Introduction

Palpation is a fundamental and crucial skill in physical examination for assessing the presence and characteristics of lesions. However, palpation techniques rely heavily on the physician’s experience and subjective sensation. Seffinger et al. reported that inter-rater reliability in palpation is not necessarily high, suggesting that objective evaluation in palpation is difficult [1].

This subjectivity directly leads to challenges in medical education. The appropriate application of force and the tactile sensations perceived by skilled physicians constitute tacit knowledge, making them extremely difficult to verbalize and convey to medical students [2]. Consequently, current training methods present a problem in that students require significant time to acquire the correct sensations. To facilitate effective skill acquisition, technical support is required to quantify the force, sensation, and motion during palpation and to provide learners with objective feedback [3].

Various sensors have been proposed to quantify palpation techniques. Probe-type sensors that measure the hardness of a target object by pressing against the affected area have been proposed [4-6]. However, since palpation involves active exploration of the affected area with the fingertips, probe-type sensors cannot reproduce or evaluate the sensations generated by actual palpation movements. To measure fingertip force on biological tissues without hindering palpation movements, finger-cot-type force sensors have been proposed [7]. However, because these sensors cover the fingertip, they present the challenge of obstructing cutaneous sensation. In soft tissue palpation, multimodal information, such as texture, temperature, and moisture, in addition to stiffness, is critical, and medical students must learn to perceive these complex sensations with their own fingers. Therefore, a structure that does not obstruct cutaneous sensation is indispensable for educational tools.

To keep the fingertip uncovered, several methods have utilized fingernail strain to estimate fingertip force [8-10]. However, these early sensors were often disposable and required cumbersome attachment procedures, making them impractical for repeated use in medical training settings. Furthermore, these methods typically failed to account for the distribution of nail strain that changes with finger posture, leading to significant errors in normal force estimation. Other challenges associated with nail strain methods included cumbersome attachment procedures, the disposable nature of the sensors, and low accuracy in estimating normal force. Additionally, sensors that measure fingertip force using skin deformation have been proposed [11]; however, accurate measurement is difficult when pressing into soft objects due to interference between the sensor and the object. Furthermore, a method combining Haplog [11] with a depth camera to measure softness has been reported [12], but this approach suffers from limitations due to the camera’s field of view and occlusion issues, which restrict free palpation movements.

Therefore, the primary objective of this study is to validate the technical performance and feasibility of a wearable sensor system designed to quantify object stiffness without obstructing fingertip sensation. By integrating nail strain sensing with inertial data for posture and displacement tracking, this study evaluates whether such a system can reliably distinguish different levels of stiffness in a simulated palpation environment using sponges of varying stiffness.

## Materials and methods

Validation strategy

This study follows the principles of medical device design validation to ensure that the developed sensor system meets the functional requirements for medical palpation training.

Scope and Objectives

The scope is limited to the technical validation of two core parameters: vertical contact force and indentation depth. The objective is to determine if the sensor provides sufficient accuracy to distinguish different levels of tissue stiffness without obstructing the user’s tactile sensation.

Acceptance Criteria

Based on previous haptic sensor studies [11] and the requirements for clinical palpation (where a force resolution of ~0.2 N is typically required to detect small nodules), we set the following acceptance criteria for this validation: 1) fingertip force estimation error (root mean square error (RMSE)) <0.2N; 2) indentation depth estimation error (RMSE) <1.0mm; and 3) statistical significance (p<0.05) in discriminating at least three different stiffness levels using the derived secant stiffness.

System design

The proposed sensor system is designed to derive object stiffness through the simultaneous measurement of fingertip force via fingernail strain gauges and finger behavior (posture and indentation depth) via an inertial measurement unit (IMU, which consists of an accelerometer and a gyroscope). This integrated approach aims to resolve the errors caused by posture changes and the movement restrictions inherent in camera-based systems.

**Figure 1:**
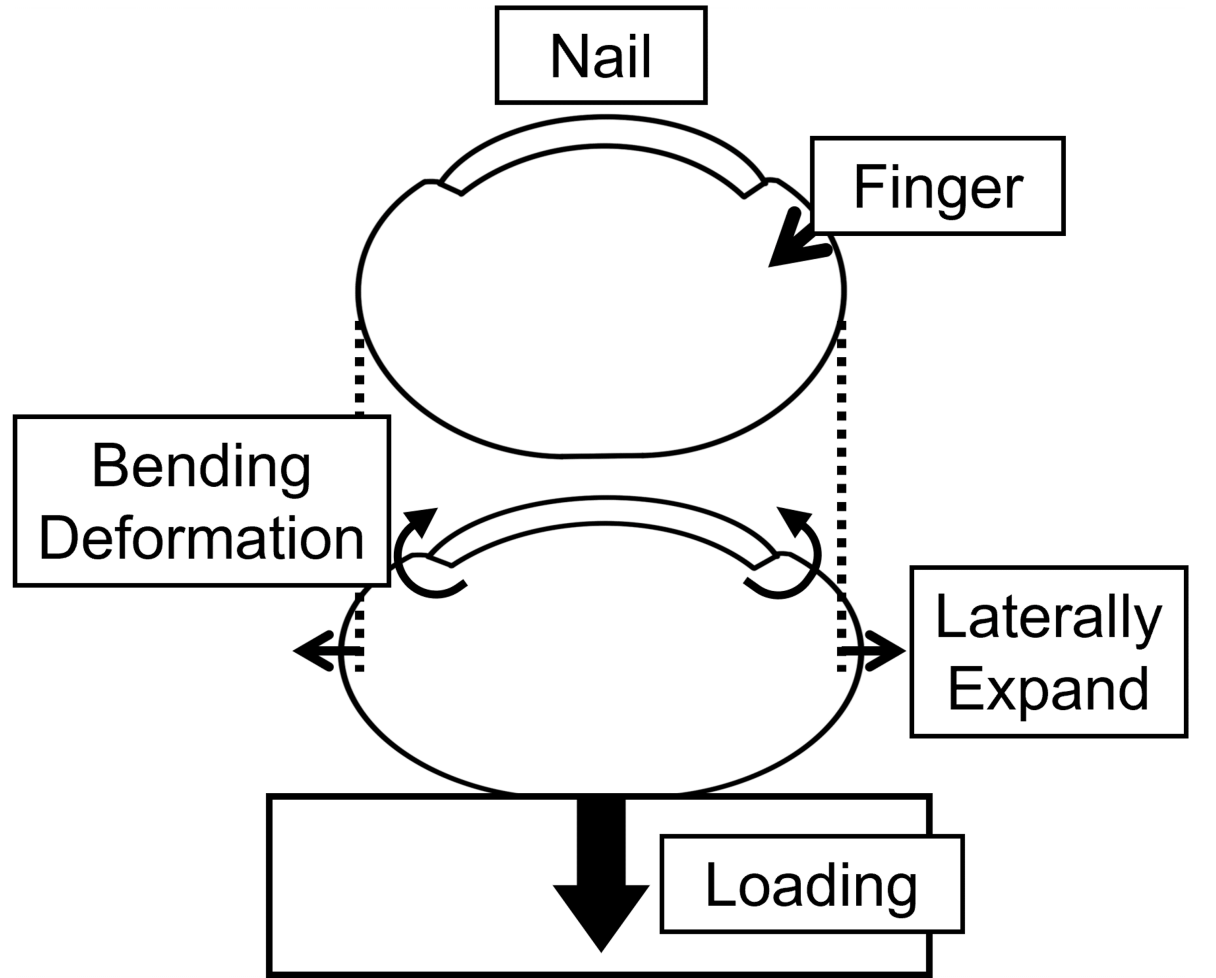
Schematic illustration of the measurement principle Fingertip force causes the skin to expand laterally, lifting the edges of the fingernail and generating strain within the nail. Image credit: Shohei Ueda (Author)

Figure 1 illustrates the principle of nail strain measurement. When the fingertip applies force to an object, the skin expands laterally. Simultaneously, the skin lifts both edges of the nail, generating strain within the nail [10,13]. Previous studies have demonstrated a correlation between fingernail strain and fingertip force [14]; therefore, fingertip force can be estimated by utilizing this strain.

**Figure 2:**
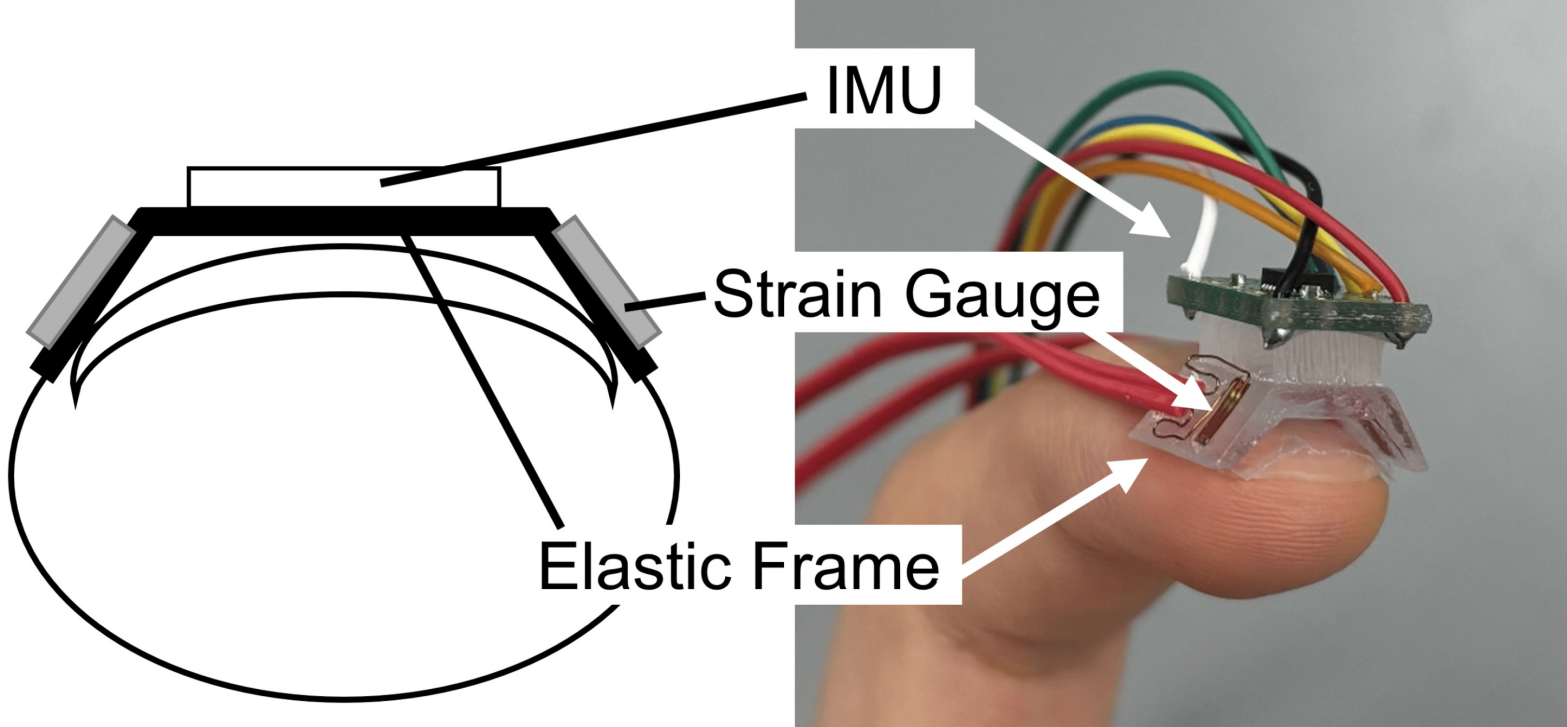
Structure of the proposed wearable softness sensor The sensor consists of a strain gauge attached to a trapezoidal elastic frame and an IMU mounted on top. IMU, inertial measurement unit Image credit: Shohei Ueda (Author)

To measure fingernail strain, we developed a sensor comprising strain gauges (KFGS-1-120-C1-11, KYOWA Electronic Instruments, Tokyo, Japan) and an elastic frame designed to transmit the nail strain to the gauges, as shown in Figure 2. The elastic frame was fabricated via 3D printing, with the 3D model designed using 3D modeling software (Blender, Blender Foundation).

In this study, we developed a sensor satisfying specific design requirements: the elastic frame must be sufficiently flexible to detect minute nail strains yet durable against breakage, and it must accommodate individual differences in nail size and curvature to a certain extent.

First, a UV-curable soft resin (“Star Drop” Soft, PADICO, Tokyo, Japan) was selected as the material for the elastic frame. This soft resin is tear-resistant and durable, even after repeated attachment and detachment. To address individual differences in nail size, seven types of elastic frames were prepared. Next, to accommodate variations in nail curvature, the elastic frame was molded into a trapezoidal arch structure. This design enables the sensor to capture deformation at both edges of the nail regardless of curvature, eliminating the need to adjust the frame shape for different users. Biocompatible double-sided tape (1522, Solventum, St. Paul, MN, USA) was used to adhere the elastic frame to the nail. Additionally, an IMU was placed on the top of the elastic frame to estimate finger posture and indentation depth.

Force estimation algorithm

As described in the previous section, there is a correlation between fingernail strain and fingertip contact force. However, Shimawaki et al. reported that fingernail strain also varies when the angle of the fingertip relative to the contact surface changes [14]. Therefore, to reduce errors in contact force estimation, it is necessary to consider finger posture information in addition to nail strain data.

When the finger posture changes, the inclination of the finger relative to the direction of gravity shifts, causing variations in the accelerometer output. In this study, assuming that the palpation motion is quasi-static, we utilized the three-axis acceleration data obtained from the IMU primarily as information representing the finger’s inclination angle.

**Figure 3:**
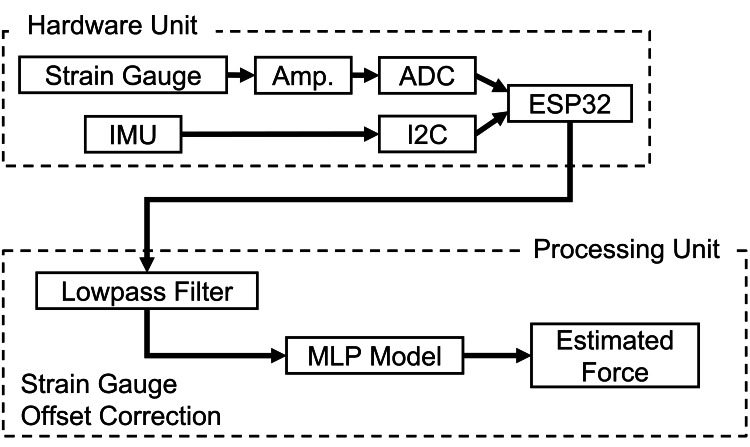
System configuration and signal processing flow for force estimation Strain and IMU data are input into an MLP to estimate fingertip force. IMU, inertial measurement unit; MLP, multilayer perceptron Image credit: Shohei Ueda (Author)

The measured strain data and the acceleration data containing posture information were input into a multilayer perceptron (MLP) neural network to estimate the contact force. The MLP model was implemented using Python (version 3.12, Python Software Foundation, Wilmington, DE, USA) with the scikit-learn library. The architecture consisted of three hidden layers with 128, 64, and 32 neurons, respectively, utilizing the rectified linear unit (ReLU) activation function and the Adam optimizer. To prevent overfitting and ensure the model's generalizability to new data, we employed a hold-out validation strategy: the model was trained solely on a separate 180-second calibration dataset, and the subsequent experimental trials were used exclusively for testing. The maximum number of iterations was set to 1,000. Figure 3 illustrates the system configuration.

Indentation depth estimation algorithm

The indentation depth of the fingertip is calculated via double integration of acceleration data obtained from the IMU. However, since the finger posture constantly changes during palpation, acceleration measured in the sensor coordinate frame cannot be integrated directly. Therefore, a coordinate transformation was performed using the following procedure.

First, a fourth-order Butterworth low-pass filter (cutoff frequency: 5 Hz) was applied to the acceleration and angular velocity data obtained from the IMU to remove sensor noise.

Next, to perform an accurate coordinate transformation, the finger posture was estimated. In this study, a quaternion $\boldsymbol{q}$ was used to represent posture in three-dimensional space, and the Madgwick Filter was applied for its estimation [15]. Here, the filter gain ${\beta}$ was set to 0.5.

Using the estimated quaternion $\boldsymbol{q}$, a rotation matrix ${R}(\boldsymbol{q})$ was derived. As shown in Equation (1), the acceleration vector $\boldsymbol{a}_s$ in the sensor coordinate frame was rotationally transformed into the acceleration vector $\boldsymbol{a}_w$ in the world coordinate frame. Here, the world coordinate system is defined such that the Z-axis aligns with the direction of gravity.

$\boldsymbol{a}_w(t) = R(\boldsymbol{q}(t)) \cdot \boldsymbol{a}_s(t) \tag{1}$

Subsequently, the linear acceleration vector $\boldsymbol{a}_{\mathrm{lin}}$, derived from the motion of the fingertip, was extracted from the transformed acceleration vector $\boldsymbol{a}_{\mathrm{w}}$ by subtracting the gravitational acceleration vector $\boldsymbol{g}=[0, 0, 9.8]^T$ using Equation (2).

$\boldsymbol{a}_{\mathrm{lin}}(t) = \boldsymbol{a}_w(t) - \boldsymbol{g} \tag{2}$

Theoretically, the indentation depth can be derived by double integrating the Z-axis component of the linear acceleration vector. In practice, however, sensor noise and posture estimation errors accumulate during integration. Without correction, this results in drift and artifacts, such as the estimated position fluctuating or moving in the reverse direction, rather than faithfully tracking the actual motion.

Therefore, considering that palpation is a reciprocating motion consisting of loading and unloading phases, the following correction processing was performed. First, motion intervals were segmented for each pushing motion based on the acceleration waveform. Second, linear trend removal was applied by assuming that the acceleration and velocity of the fingertip were zero at the start and end of each motion interval (valid for typical discrete palpation). Before integration, the linear trend was subtracted from the acceleration waveform to correct the values at both ends of the interval to zero; furthermore, a similar linear correction was applied to the velocity after integration to reset the accumulated error.

Finally, the indentation depth of the fingertip was calculated by performing numerical integration using the trapezoidal rule on the acceleration and velocity corrected by the above processes.

Softness estimation algorithm

Object softness was quantified using the fingertip force ${F}$ obtained from the nail strain sensor and the indentation depth ${d}$ obtained from the IMU. Generally, Young's modulus ${E}$ is used as a physical quantity to represent the softness of a material. Derivation of Young's modulus requires information on stress $\sigma$ and strain $\varepsilon$, as shown in Equation (3).

$E = \frac{\sigma}{\varepsilon} \tag{3}$

Here, as shown in Equation (4), stress $\sigma$ is derived from the load $F$ applied to the object and the contact area, while strain $\varepsilon$ is derived from the displacement $d$ and the thickness $L$ of the object.

$\sigma = \frac{F}{A}, \quad \varepsilon = \frac{d}{L} \tag{4}$

In actual palpation movements, the contact area $A$ between the fingertip and the object changes non-linearly with indentation. Furthermore, the thickness $L$ of the object, such as biological tissue, is often unknown. Therefore, it is difficult to accurately estimate Young's modulus using only wearable sensors. On the other hand, Stiffness $K$ serves as an index representing the structural hardness of an object. Stiffness $K$ is defined as the relationship between load $F$ and displacement $d$, as shown in Equation (5).

$K = \frac{F}{d} \tag{5}$

Stiffness can be derived from the force applied by the fingertip and the indentation depth, even if the contact area and object thickness are unknown. Therefore, in this study, we calculated the slope of the line connecting the origin to the maximum displacement point during the loading phase of the obtained force-displacement curve. This value was defined as the stiffness value $K$ [N/mm] and used as the index of softness.

Experiments

Force Accuracy Validation

**Figure 4:**
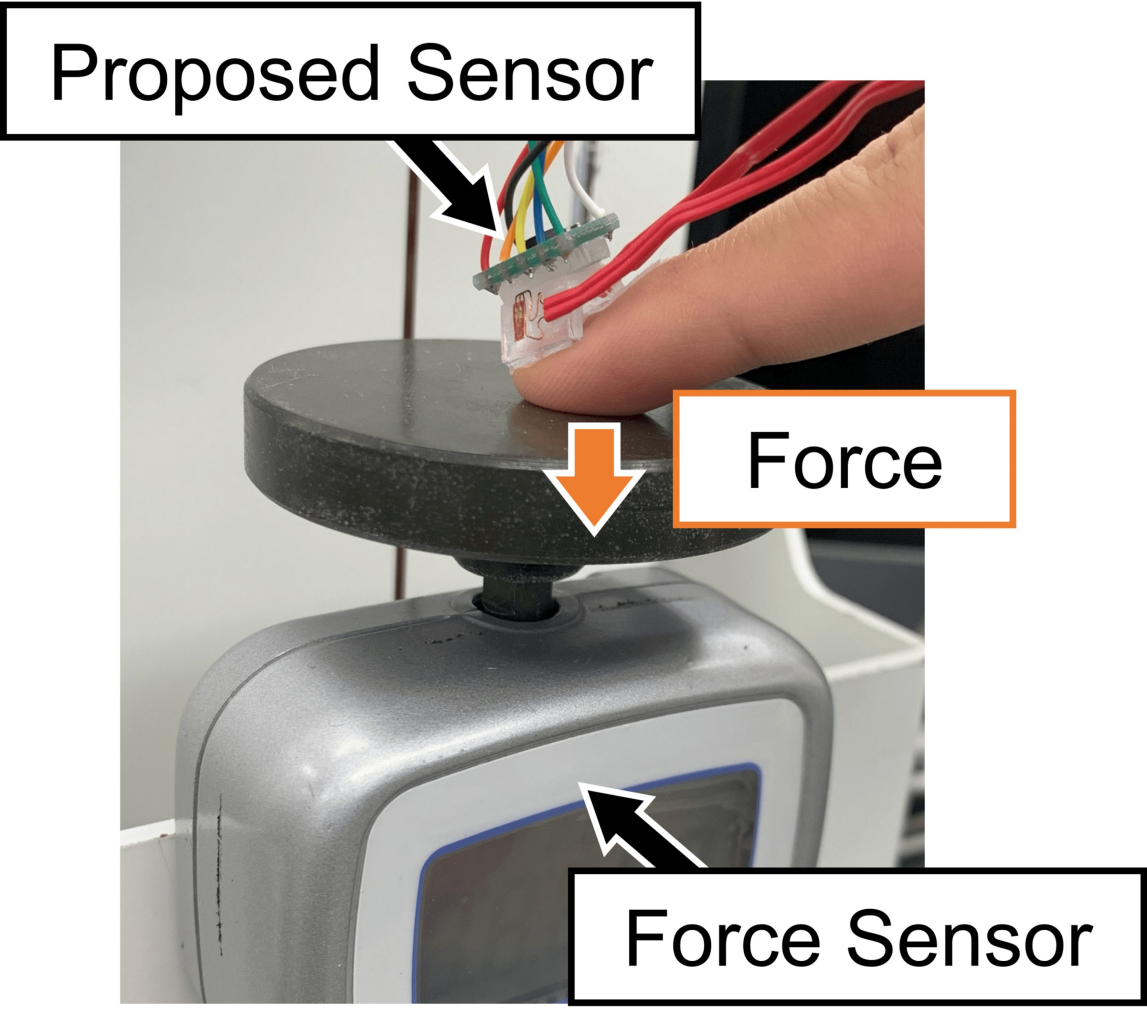
Experimental setup for force accuracy validation The participant applied force to a digital force gauge using the index finger equipped with the sensor. Image credit: Shohei Ueda (Author)

An experiment was conducted with one subject to evaluate the estimation accuracy of the contact force. The subject wore the proposed sensor on the index fingernail and performed a motion of applying force to a digital force gauge (FGP-10, Nidec-Shimpo, Kyoto, Japan) 10 times across five trials, as shown in Figure 4. A total of 50 force application motions were performed. The motions were executed at intervals of 1 second.

To perform estimation using a neural network, training data were acquired through calibration. Mascaro et al. [8] proposed a calibration trajectory based on a friction cone model to estimate the shear and normal forces of the fingertip from fingernail strain. However, in the estimation results reported by Mascaro et al., the accuracy of normal force estimation was lower than that of shear force. This is presumably because their calibration motion involved minimal load variation in the normal direction. Therefore, in this study, calibration was performed using a motion that incorporated a pattern of increasing and decreasing normal force into the calibration trajectory proposed by Mascaro et al.

Displacement Accuracy Validation

To verify the geometric accuracy of the displacement estimation algorithm, a validation experiment was conducted using an experimental apparatus constrained to uniaxial motion to eliminate disturbances such as irregular hand tremors.

**Figure 5:**
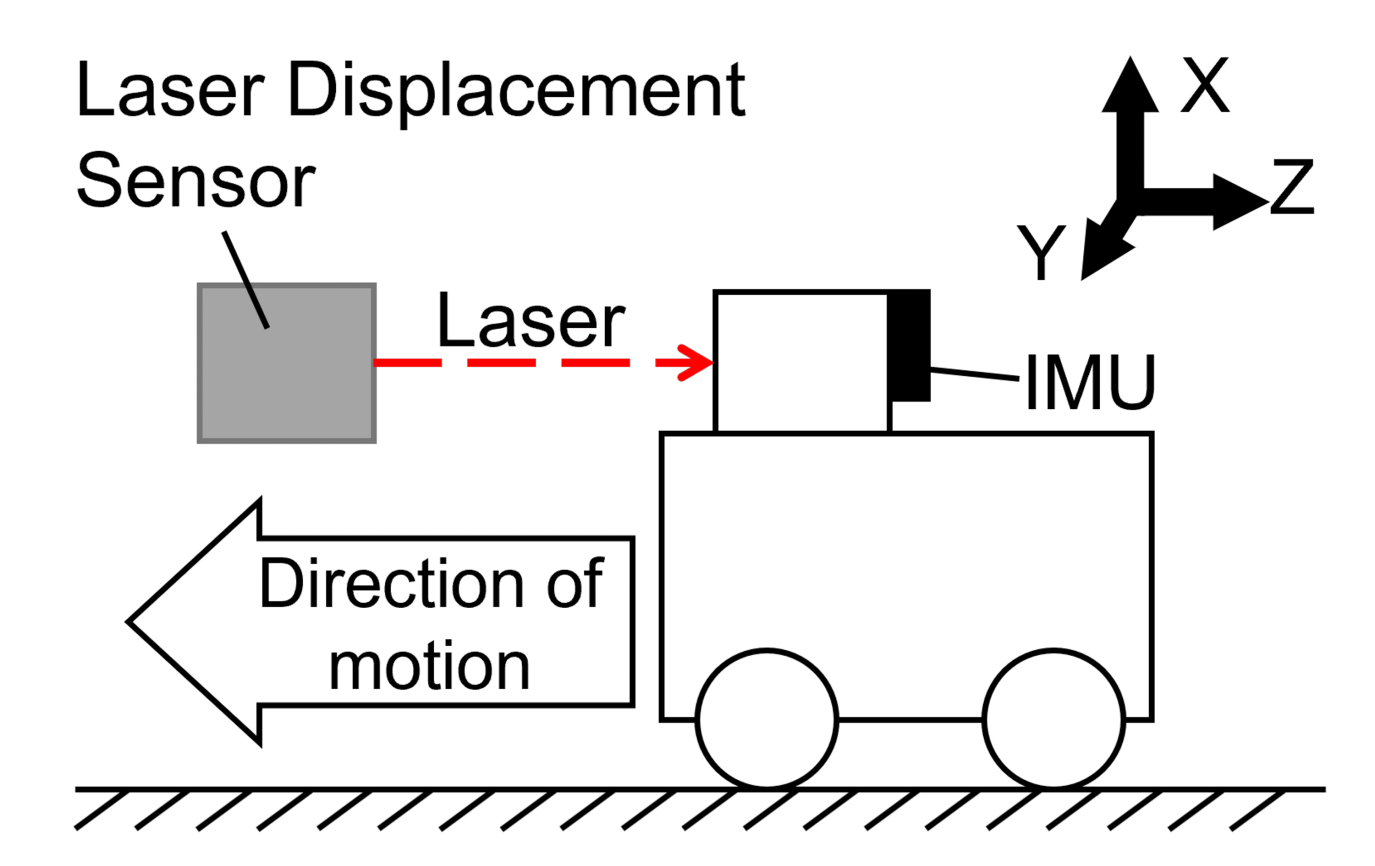
Experimental setup for displacement accuracy validation An IMU was mounted on a linear slider constrained to uniaxial motion to exclude hand tremors. A laser displacement sensor was used to measure the ground truth. IMU, inertial measurement unit Image credit: Shohei Ueda (Author)

As shown in Figure 5, an IMU (ICM-20948, TDK InvenSense, San Jose, CA, USA) was fixed to the apparatus, and linear motion was manually performed five times. During this process, the displacement of the apparatus was measured using a laser displacement sensor (CD22-100VM122, OPTEX FA, Kyoto, Japan) to serve as the ground truth. The sampling frequency for both the IMU and the laser displacement sensor was set to 200 Hz.

Softness Quantification Experiment

To verify the softness discrimination capability of the proposed sensor, three types of sponges with different softness levels (soft, medium, and hard) were prepared. The dimensions of each sample were 20×30×30 mm. The experiment was conducted with one healthy adult male subject.

To measure the stiffness value of each sample, a compression test was performed using a digital force gauge (FGP-10, Nidec-Shimpo) equipped with a 12 mm diameter indenter on its tip. The size of this indenter was selected to simulate the contact area of a human fingertip.

**Figure 6:**
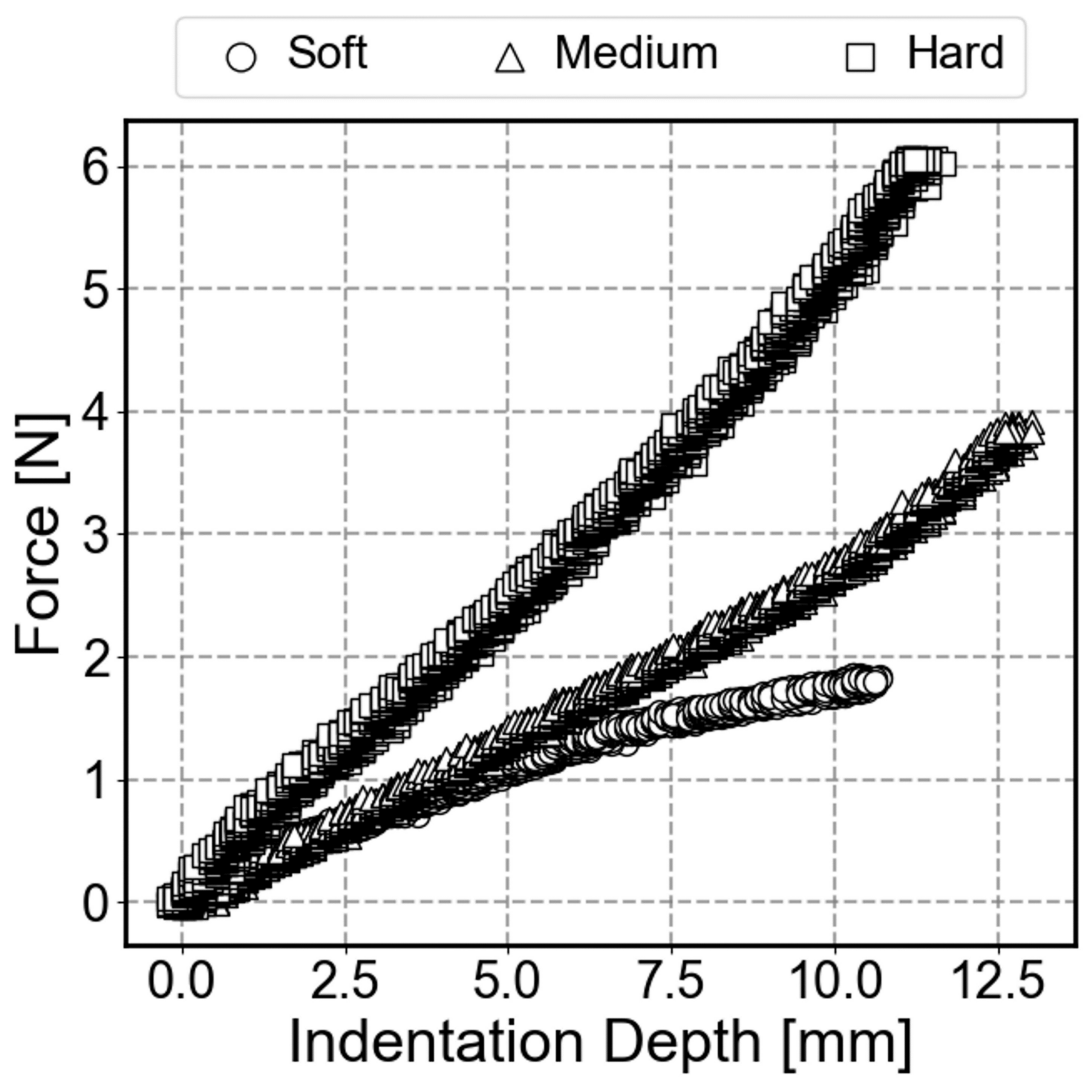
Reference force-displacement curves obtained from compression tests A digital force gauge with a 12 mm diameter indenter was used. The reference stiffness values were calculated using secant stiffness at the maximum load.

Figure 6 shows the relationship between force and displacement during loading obtained from the compression test. As a result of performing linear regression on the force-displacement curve, the reference stiffness values for the samples were soft: 0.17 N/mm, medium: 0.32 N/mm, and hard: 0.52 N/mm, respectively.

**Figure 7:**
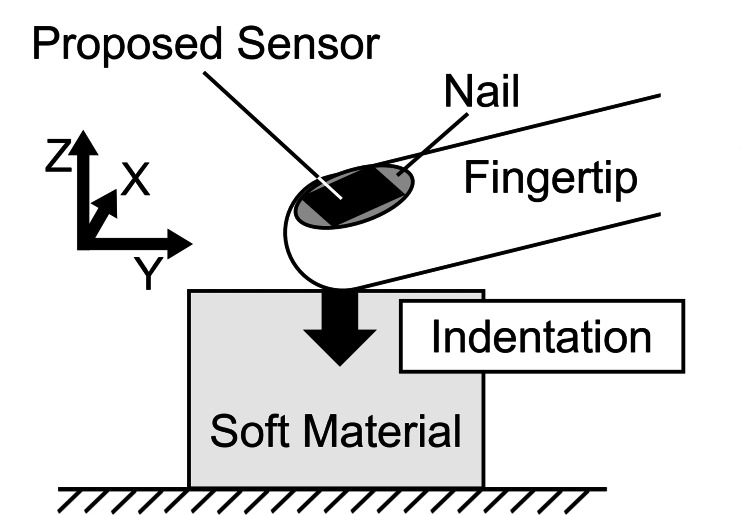
Experimental setup for softness quantification The participant performed indentation on three types of sponges (soft, medium, and hard). Image credit: Shohei Ueda (Author)

In the softness measurement experiment using the proposed sensor, the subject performed 10 consecutive pushing motions on each sample, as shown in Figure 7. Additionally, to ensure the accurate functioning of the zero correction for acceleration and velocity within the integration interval of the IMU output, a stationary period of approximately 1 second was provided between the loading and unloading phases.

Statistical analysis

Data analysis was conducted to evaluate the sensor's accuracy and its ability to discriminate stiffness. We used Welch’s one-way ANOVA to compare the stiffness values among the three groups, as the assumption of homogeneity of variances was violated (Levene’s test, p<0.05). For post-hoc comparisons, the Games-Howell test was performed. All analyses were conducted using Python (version 3.12, Python Software Foundation, Wilmington, DE, USA) with the Pingouin and SciPy libraries. The level of significance was set at $\alpha = 0.05$.

## Results

Force estimation

**Figure 8:**
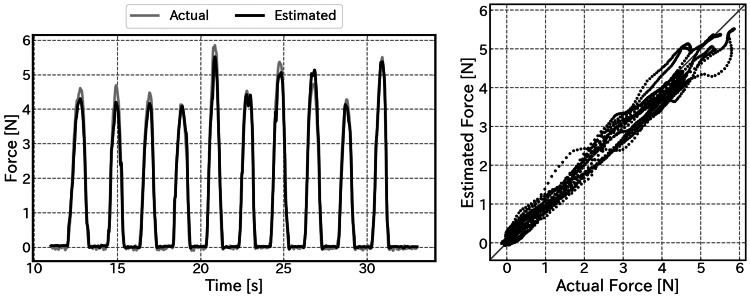
Results of fingertip force estimation (Left) Time-series comparison between the estimated force (black line) and actual force (gray line). (Right) Scatter plot showing the linearity of the estimation.

Figure 8 presents a representative example of fingertip force estimation using the fingernail strain sensor. The time-series graph demonstrates that the estimated values track the fluctuations in the ground truth values with negligible latency, accurately capturing peak values even during rapid load changes.

Furthermore, the scatter plot illustrating the relationship between the ground truth and estimated values reveals that the data points are distributed along the ideal line, confirming high linearity. The Pearson correlation coefficient () was 0.997, demonstrating excellent agreement between the estimated and actual force.

Hysteresis was quantitatively evaluated by calculating the maximum difference in estimated force between the loading and unloading phases for each palpation cycle. The average maximum hysteresis error across five trials was 0.54 N, which corresponds to approximately 12.8% of the maximum applied force.

**Figure 9:**
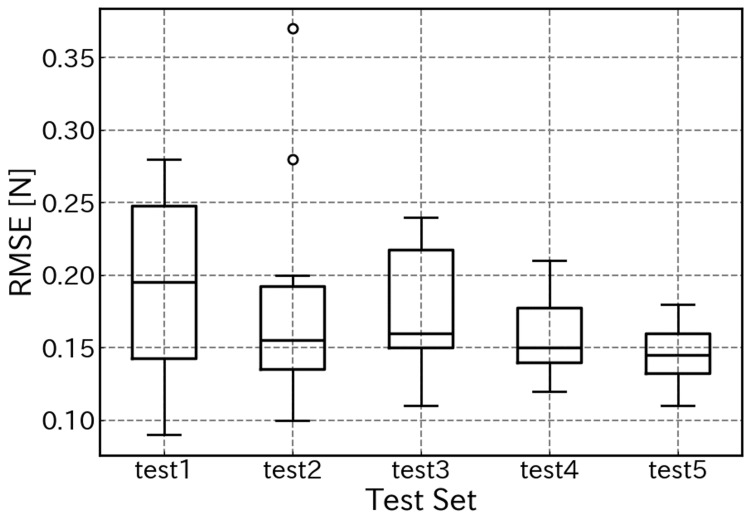
Box plot of the RMSE for force estimation across five trials RMSE, root mean square error

Figure 9 displays a box plot of the RMSE across all five trials (a total of 50 pushing motions). The average RMSE across all trials was 0.17±0.02 N. This corresponds to approximately 4.1% of the maximum applied force, indicating sufficient accuracy for distinguishing tissue stiffness in clinical palpation. The median value for each trial was consistently below 0.2 N, indicating low variability in performance between trials and demonstrating high reproducibility. Although outliers were observed in some trials, stable estimation accuracy was maintained overall.

Displacement estimation

**Figure 10:**
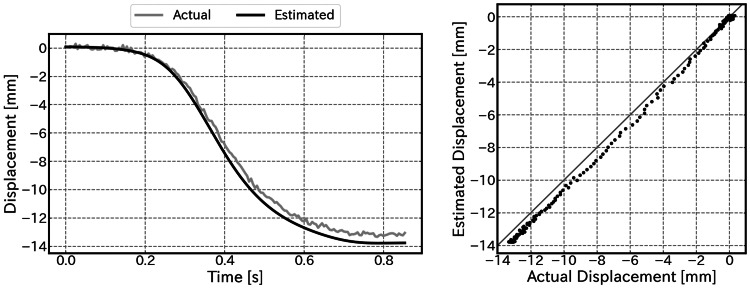
Results of displacement estimation (Left) Time-series comparison between the estimated displacement (black line) and actual displacement (gray line). (Right) Scatter plot showing the linearity.

Figure 10 shows an example of the displacement estimation results derived by applying coordinate transformation and integration processes to the acquired IMU data. In the time-series graph, the estimated values track the ground truth values well. Although a slight deviation due to the accumulation of integration errors is observed as the displacement approaches the maximum, the shape of the waveform is correctly captured from the start to the end of the motion. Similarly, in the scatter plot, the data points are distributed along the ideal line, demonstrating good linearity.

Table 1 presents the maximum displacement values (calculated vs. ground truth) and errors for each trial. The average RMSE across all five trials was 0.75±0.31 mm. The average error at the terminal point was -0.14±0.69 mm; this value is close to zero, confirming that there is no specific bias in the displacement estimation algorithm. The average absolute error was 0.58±0.29 mm, indicating low variability between trials and demonstrating that stable displacement estimation is possible.

**Table 1:** RMSE and terminal error of displacement estimation for each trial Terminal error is defined as the difference between the estimated value and the ground truth at the maximum displacement. RMSE, root mean square error

Trial	Estimated value (mm)	Ground truth (mm)	Terminal error (mm)	RMSE (mm)
1	-13.78	-13.34	-0.44	0.49
2	-16.06	-15.00	-1.06	1.18
3	-14.03	-13.73	-0.30	0.40
4	-18.47	-19.07	0.59	0.89
5	-20.09	-20.60	0.51	0.78

Softness quantification

**Figure 11:**
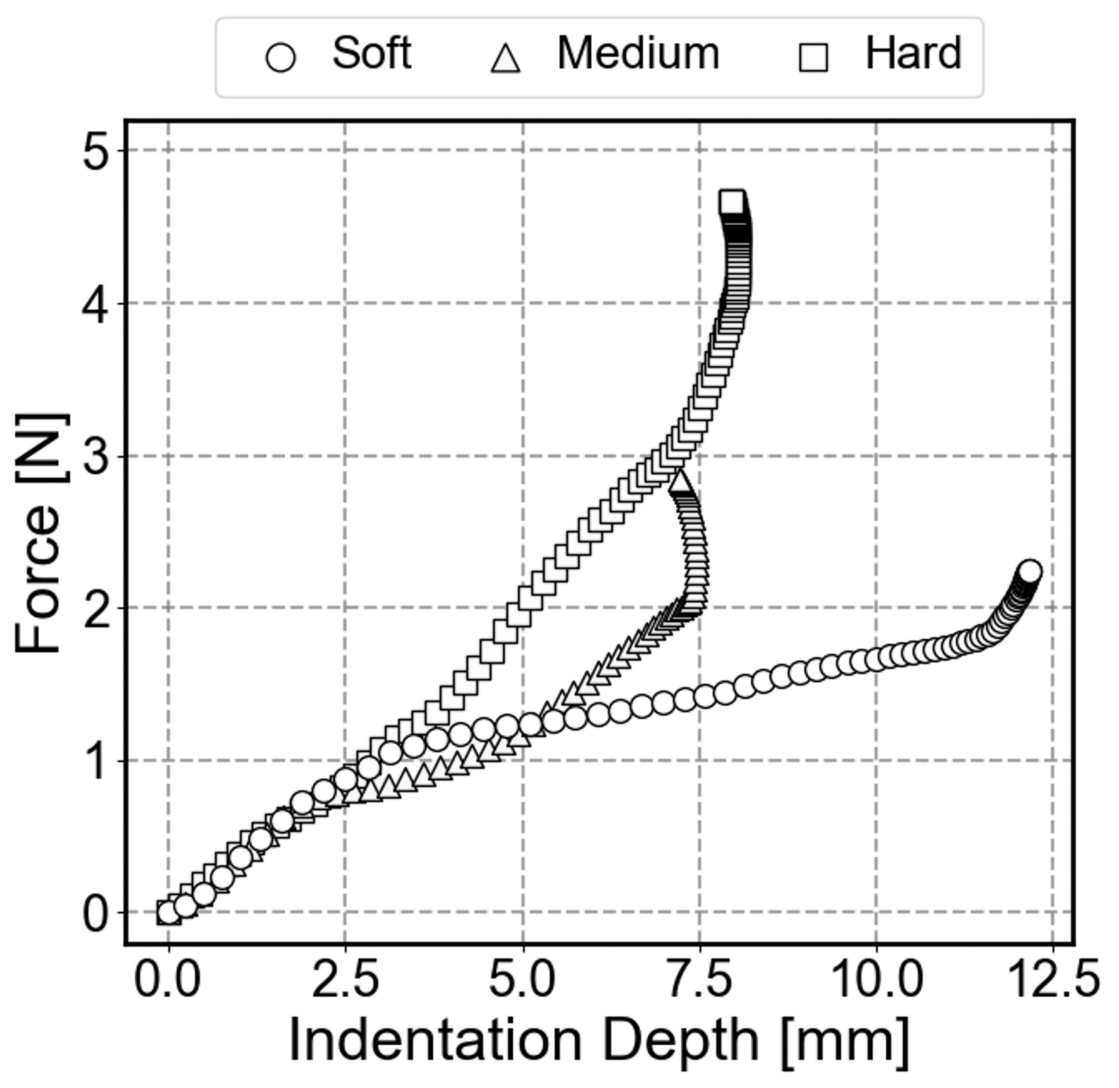
Representative force-displacement curves for three sponge samples estimated by the proposed sensor The slope of the curve indicates the stiffness of the object.

Figure 11 displays representative force-displacement curves for each sample. The slope of the graph increases in the order of Soft, Medium, and Hard, confirming that the proposed sensor captures the differences in object softness.

**Figure 12:**
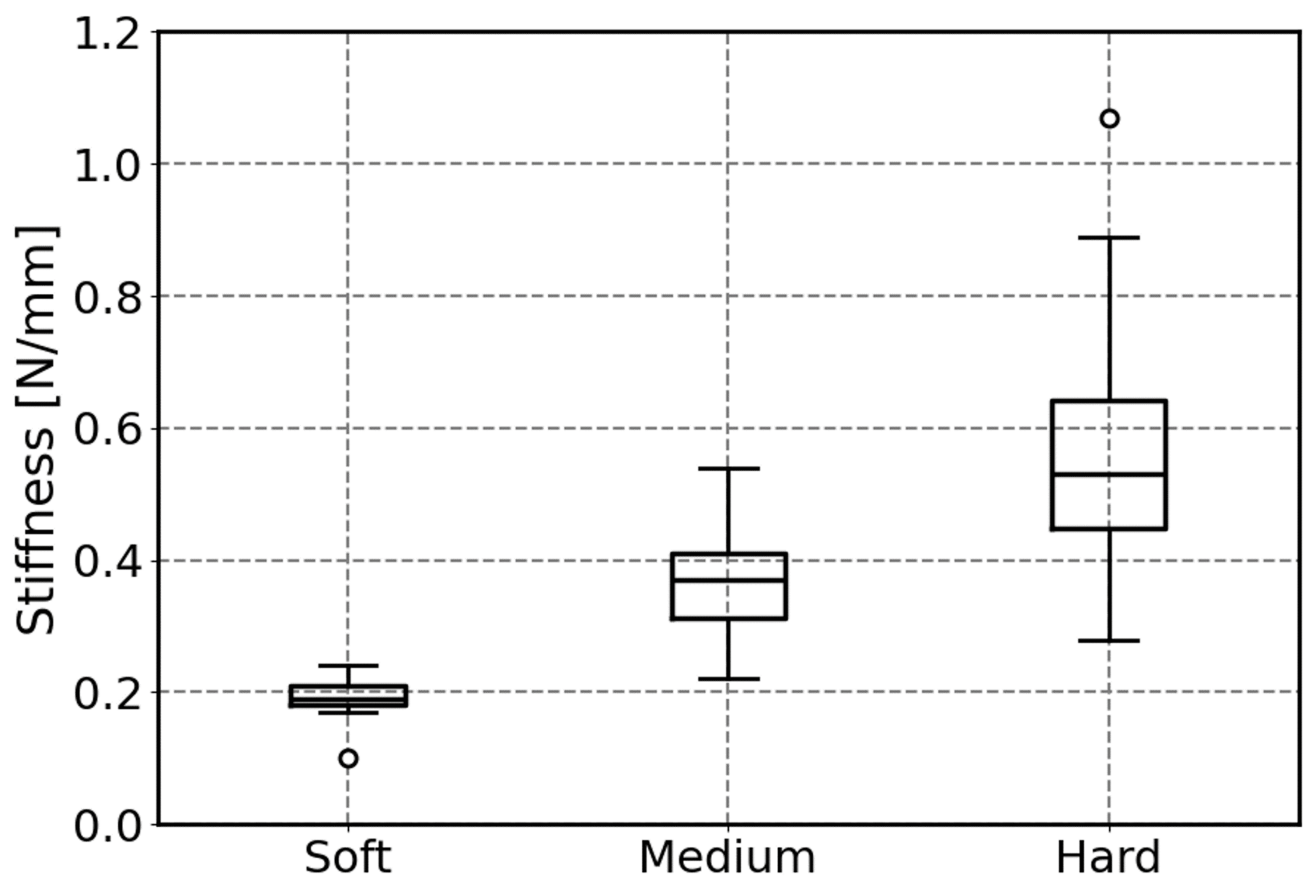
Box plot of the estimated stiffness values for three sponge types Data were collected from 30 trials (10 trials per sponge). Statistical analysis was performed using Welch’s one-way ANOVA followed by the Games-Howell post-hoc test. The ANOVA revealed a significant difference among the three groups (F(2,13.98)=26.86, p<0.001). The Games-Howell test showed significant differences for all pairs: soft vs. medium (p<0.001), soft vs. hard (p=0.001), and medium vs. hard (p=0.045).

Figure 12 presents a box plot of the stiffness values calculated from a total of 30 trials (three types×10 trials). The average stiffness values were 0.19 N/mm for soft, 0.37 N/mm for medium, and 0.59 N/mm for hard. Although a trend toward larger data variability was observed for the hard sample, the interquartile ranges for each group did not overlap, confirming clear separation. It should be noted that these stiffness values were derived from repeated trials by a single participant. Therefore, the reported variability reflects intra-operator variability (reproducibility of the system under a single user) rather than inter-operator differences.

To statistically evaluate the discriminative capability of the sensor, Welch’s one-way ANOVA was performed on the stiffness values, as equal variances could not be assumed. The results indicated a significant difference among the three groups (F(2, 13.98)=26.86, p<0.001), with a large effect size (partial ). Furthermore, post-hoc comparisons using the Games-Howell method revealed statistically significant differences (p<0.05) for all pairs (soft vs. medium, soft vs. hard, and medium vs. hard). These results demonstrate that the proposed sensor can significantly discriminate between three different levels of softness.

## Discussion

In this study, we developed a wearable softness sensor that does not obstruct tactile sensation, aiming to quantify palpation skills and facilitate their application in medical education. The proposed sensor system consists of a fingernail strain sensor for fingertip force estimation and an IMU for displacement estimation.

The accuracy of both fingertip force estimation and displacement estimation was verified. Experimental results demonstrated that the developed fingernail strain sensor achieved high linearity and reproducibility regarding fingertip force by incorporating posture correction via an MLP. Although a hysteresis error of approximately 12.8% was observed, this is largely attributed to the inherent viscoelastic properties of the human fingertip and fingernail rather than the sensor mechanics itself. Since human tactile perception also exhibits hysteresis, this behavior is considered acceptable for reproducing the sensations involved in palpation. Furthermore, regarding displacement estimation using the IMU, it was shown that displacement could be estimated with high accuracy by suppressing the accumulated error associated with integration through linear trend removal and zero-velocity updates. These findings indicate that force and displacement can be measured simultaneously using only wearable sensors, without relying on external cameras or probe-type sensors.

In experiments using three types of sponges, the proposed sensor successfully and significantly distinguished differences in object stiffness. The stiffness values estimated by the proposed sensor (soft: 0.19, medium: 0.37, hard: 0.59 N/mm) matched closely with the reference values measured using a force gauge and a fingertip-simulating indenter (soft: 0.17, medium: 0.32, hard: 0.52 N/mm). This suggests that the proposed method possesses the capability not only to determine the relative order of stiffness but also to quantitatively estimate the physical stiffness values of the target objects (quantitative estimation capability).

Conversely, a trend was observed wherein the variance in stiffness values increased for harder samples. This is likely because harder samples result in smaller indentation depths, which serve as the denominator in the stiffness calculation, thereby causing the displacement estimation error to become relatively larger. However, even with this increased variance, the samples were statistically clearly distinguishable from one another.

Beyond the numerical stiffness values, the system's ability to capture dynamic mechanical properties is crucial. While this study utilized sponges for initial validation, biological tissues exhibit more complex, nonlinear, and viscoelastic behaviors compared to synthetic materials. In clinical settings, the force-displacement relationship often shows significant hysteresis and stress relaxation. Importantly, the proposed sensor system records force and displacement as continuous time-series data, allowing for the capture of such dynamic characteristics. As observed in Figure 11, the sensor successfully captured the nonlinear force-displacement profiles (convex curves) of the sponges, suggesting that it possesses the capability to quantify the viscoelastic properties of real biological tissues in future clinical applications.

The primary advantage of the proposed sensor is that the finger pad remains completely unobstructed. This allows medical students to directly perceive the texture and temperature of the object while simultaneously confirming their applied force, indentation depth, and tactile sensation of softness as objective numerical data. In palpation education, which has traditionally relied on the instructor's experience, intuition, and tacit knowledge, the introduction of such objective metrics is expected to contribute significantly to shortening the learning curve and standardizing techniques.

This study has several limitations. First, this experiment is a proof-of-concept study conducted with one subject. Since there are individual differences in nail size, curvature, and finger shape, large-scale subject experiments are required to verify the generalizability of the proposed method. Second, the number of soft object samples used was limited. In actual palpation, it is necessary to distinguish subtle differences in the stiffness of biological tissues. To verify the resolution of softness distinguishable by the proposed method, further experiments involving a larger number of samples are necessary.

## Conclusions

In this study, we proposed an unobstructed wearable softness sensor utilizing fingernail strain and an IMU, aiming for applications in palpation training for medical education. Evaluation experiments confirmed that, unlike existing finger-covering sensors or camera-based methods, the proposed sensor enables high-precision estimation of fingertip force and indentation depth during natural palpation movements. Additionally, the sensor demonstrated the ability to significantly discriminate stiffness differences among three sponge types with varying levels of softness.

This technology holds promise as a novel educational tool capable of formalizing the palpation skills of skilled physicians and providing objective feedback to medical students. Future work will involve validation with a larger cohort of subjects and the application of the system to the measurement of biological tissues.
